# A finger-jointing model for describing ultrastructures of cellulose microfibrils

**DOI:** 10.1038/s41598-021-89435-6

**Published:** 2021-05-12

**Authors:** Bunshi Fugetsu, Vipin Adavan Kiliyankil, Shoichi Takiguchi, Ichiro Sakata, Morinobu Endo

**Affiliations:** 1 Institute for Future Initiatives, The University of Tokyo, Bunkyo-ku, Yayoi 2-11-16, Tokyo, 113-8656 Japan; 2 Faculty of Engineering, The University of Tokyo, Bunkyo-ku, Yayoi 2-11-16, Tokyo, 113-8656 Japan; 3 Institute of Carbon Science and Technology, Interdisciplinary Cluster for Cutting Edge Research, Shinshu University, 4-17-1, Wakasato, Nagano, 380-8553 Japan

**Keywords:** Chemistry, Materials science, Nanoscience and technology

## Abstract

In this paper, we propose a finger-jointing model to describe the possible ultrastructures of cellulose microfibrils based on new observations obtained through heating of 2,2,6,6-tetramethylpiperidine-1-oxyl (TEMPO) oxidized cellulose nanofibrils (CNFs) in saturated water vapor. We heated the micrometers-long TEMPO-CNFs in saturated water vapor (≥ 120 °C, ≥ 0.2 MPa) and observed a surprising fact that the long TEMPO-CNFs unzipped into short (100 s of nanometers long) fibers. We characterized the heated TEMPO-CNFs using X-ray diffraction (XRD) and observed the XRD patterns were in consistent with Iβ. We observed also jointed ultrastructures on the heated TEMPO-CNFs via high-resolution transmission electron microscopy (HR-TEM). Thus we concluded that cellulose microfibrils are not seamlessly long structures, but serial jointed structures of shorter blocks. Polysaccharide chains of the short blocks organized in Iβ. The jointed region can be either Iα or amorphous, depending on positions and distances among the chains jointed in proximity. Under heating, Iα was not converted into Iβ but was simply destroyed. The jointed structure implies a “working and resting rhythm” in the biosynthesis of cellulose.

## Introduction

Plant cellulose is the most abundant biomass on Earth and is synthesized via photo-synthesis with atmospheric carbon dioxide as the starting material. Full utilization of plant cellulose, therefore, is key for achieving the goal of decarbonization. The native plant cellulose appears as fibers approximately 1–3 mm in length and 30 μm in width. This macro-sized fiber exhibits drastic changes in both its physiochemical and mechanical properties when split down to nano-sized fibers. Use of 2,2,6,6-tetramethylpiperidine-1-1oxyl (TEMPO) as a catalyst to regioselectively oxidize C6 primary hydroxyls to C6 carboxylates is a standard method^1^, among others^2–5^, for splitting the native cellulose down to nano-sized fibers. The nano-sized cellulose fibers prepared via TEMPO-oxidation (TEMPO-CNFs) remain the true fiber-shape with a width of approximately 3 nm and a length of few micrometers^6^. Thin films made up entirely of TEMPO-CNFs are highly transparent and showing excellent performances in tensile strength (200–300 MPa)^6^, elastic moduli (6–7 GPa), and thermal expansion coefficiencies (2.7 ppm K^−1^)^7^.

TEMPO-CNFs have recently been produced in industrial quantities^8,9^ and an attempt to replace traditional carbon blacks in rubber industries is one of large-scaled utilizations of TEMPO-CNFs^10^.

As additives in rubber, also be a component of a certain functioning material, TEMPO-CNFs will undergo a long process of tempering. However, experimental data on stabilities of TEMPO-CNFs under tougher conditions of utilizations, especially under the conditions of high temperatures and/or high pressures, are seriously lack, due mainly to the complexity of the ultrastructures of TEMPO-CNFs.

TEMPO-CNFs can be produced from a variety of native cellulose, for example, from wood pulp, cotton, and tunicin, to bacterial cellulose^1^ but in case of the commercially available TEMPO-CNFs, cellulose from higher plants were the entire precursors. Ultrastructures of the plant cellulose have been investigated longer than a century and important milestones have been achieved. The followings are just for examples:

(i) native cellulose is a composite consisting of two distinct crystalline forms: Iɑ form and Iβ form^11^;
(ii) Iβ is a two-chain packed monoclinic unit cell whereas Iɑ is a single-chain packed triclinic unit cell, and the polysaccharide chains in both Iɑ and Iβ organized in a parallel fashion of chain packing^12,13^;
(iii) Iɑ is metastable and can be converted into Iβ by annealing in saturated steam at temperatures > 200 °C^14^;
(iv) and moreover, one individual microfibril is consisting of 24–36 chains and the axial disorder lies on microfibril twisting^15,16^.

However, significant questions remained with respect to relations between Iɑ and Iβ. These include linkages between the crystalline regions and the amorphous regions and the linkages between the two crystalline forms.

We heated the micrometers-long TEMPO-CNFs in saturated water vapor (temperature ≥ 120 °C, pressure ≥ 0.2 MPa) and observed a surprising fact that the long TEMPO-CNFs unzipped into short lengths of nanofibers (100 s of nanometers long with widths of 1.5–3.0 nm). XRD patterns were in consistent with Iβ cellulose, indicating a change on the ultrastructures of TEMPO-CNFs. We also observed jointed ultrastructures on the heated TEMPO-CNFs by using high-resolution transmission electron microscopy (HR-TEM). We proposed a figure-jointing model, in this paper, for describing the possible ultrastructures of cellulose microfibrils with the emphasis on relations between Iα and Iβ. Our finger-jointing model is also applicable for explaining the origins of the amorphous regions.

## Results and discussions

**Figure 1:**
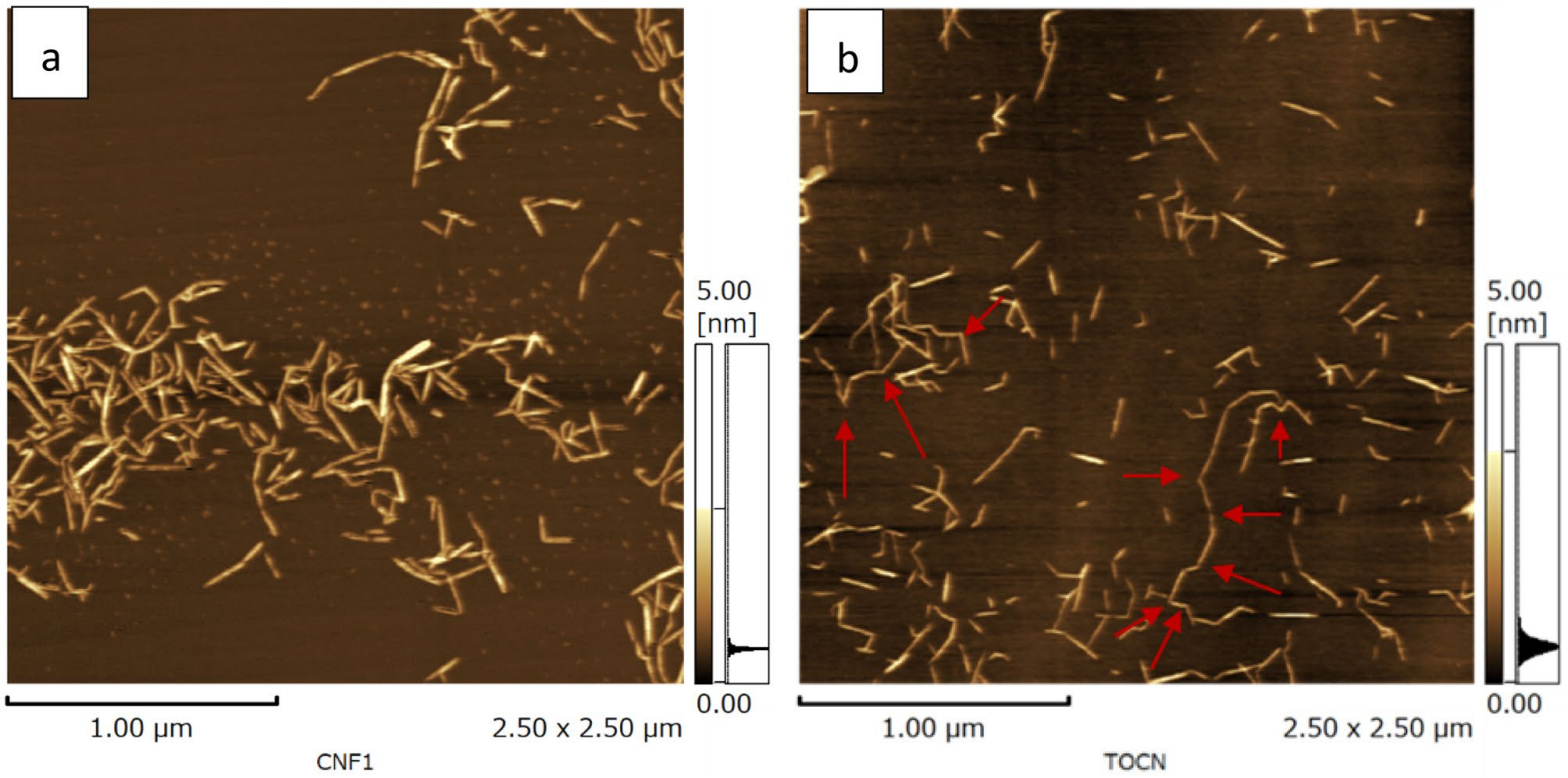
AFM images of TEMPO-CNFs after heated. The sample was prepared by heating an aqueous suspension containing 0.2 wt% of the as-produced TEMPO-CNFs in 150 °C saturated water vapor for 2 or 4 h. The samples were diluted 1/1000 with 80% ethanol. After 4 h of heating, the as-produced TEMPO-CNFs unzipped into shorter lengths of fibers (**a**); after 2 h of heating, both short and long lengths fibers were observed (**b**). The long length fibers curved and twisted (pointed by red arrows).

An aqueous suspension containing 0.2 wt% of the as-produced TEMPO-CNFs was heated in 150 °C saturated water vapor for 4 h. This heated suspension, after being cooled down to room temperatures, was observed using atomic force microscopy (AFM), Fig. 1a shows the typical AFM images. As can be seen from Fig. 1a, the micrometers long TEMPO-CNFs unzipped into shorter length of nanofibers. Thirty the short nanofibers were randomly selected and the length and the width measured. The short nanofibers had lengths of 200–600 nm and widths of 1.5–3.0 nm.

TEMPO-CNFs changed partly or completely into short nanofibers, depended on temperatures of the saturated water vapor and the time of heating the samples. Figure 1b shows typical AFM images of the same sample as in Fig. 1a but was heated in 150 °C saturated vapor for 2 h. We observed abundant short nanofibers with lengths < 600 nm and few long lengths nanofibers. It is notable that the long lengths nanofibers curved and twisted (marked with red arrows in Fig. 1b).

**Figure 2:**
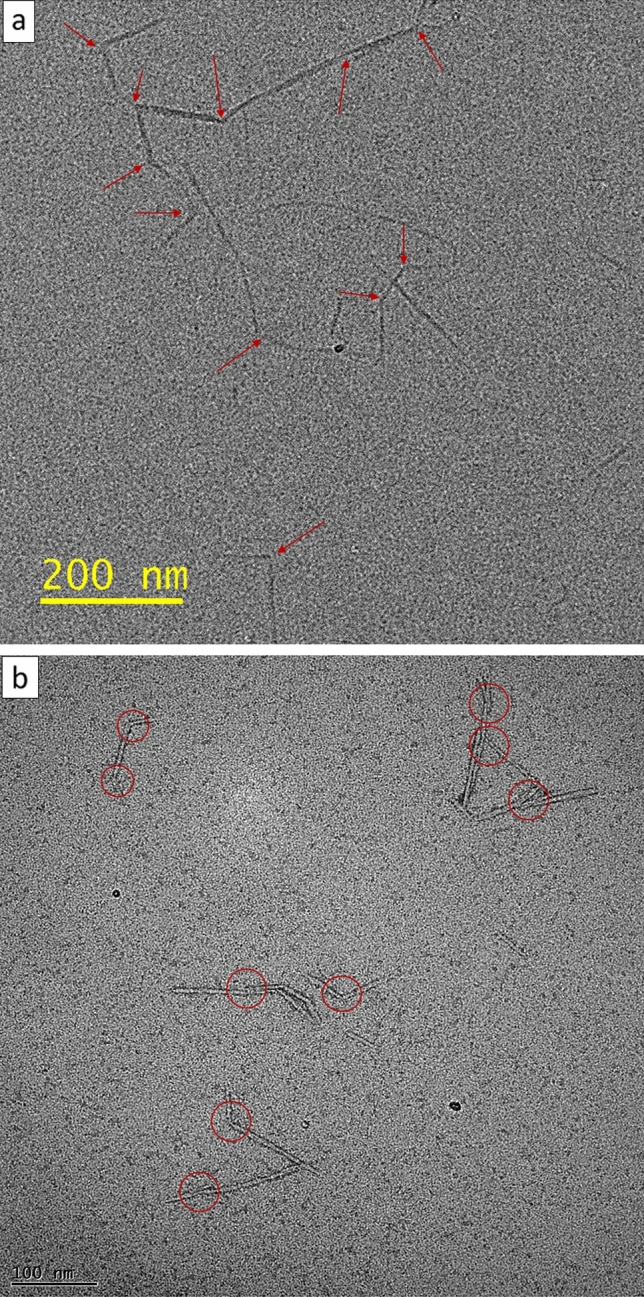
(**a**) HR-TEM images (without staining but under cooling) of TEMPO-CNFs after heated in 150 °C saturated water vapor for 2 h. We observed TEMPO-CNFs curved and twisted (pointed by red arrows). (**b**) HR-TEM images (negatively stained with 2.0% uranyl acetate) of TEMPO-CNFs after heated in 150 °C saturated water vapor for 4 h. We observed jointed ultrastructures on the heated TEMPO-CNFs (pointed by red circles).

The heated TEMPO-CNFs were analyzed by using HR-TEM in duplicate (with and without staining). Figure 2a,b show the typical images. In Fig. 2a, the heated TEMPO-CNFs were directly analyzed (without staining) under cooling via nitrogen. Again, we observed the heated TEMPO-CNFs curved and twisted (marked with red arrows). In Fig. 2b the heated TEMPO-CNFs were negatively stained via a 2% uranyl acetate solution and were then analyzed. We observed jointed ultrastructures on the heated TEMPO-CNFs (marked with red circles).

Temperatures of saturated water vapor and the time of heating samples are the key parameters affecting the morphology of TEMPO-CNFs. Under heating conditions of 120 °C/6 h, 130 °C/4 h, 140 °C/2 h, and 150 °C/1 h, the heated aqueous TEMPO-CNF suspensions are nearly colorless or lightly yellowish and highly transparent. Under heating conditions of 150 °C/4 h, 160 °C/3 h, and 180 °C/2 h, the heated aqueous TEMPO-CNF suspensions are dark brown colors and they become hydrogels under heating conditions of 150 °C/6 h, 160 °C/5 h, and 180 °C/4 h. A photo of four heated aqueous TEMPO-CNF suspensions, after cooled down to room temperatures, is given in the supporting information (Fig. S1); we heated the aqueous TEMPO-CNF suspension for 2 h, 3 h, 4.5 h, and 5 h at 140 °C, respectively, in saturated water vapor. Figure S2 shows a photo of the aqueous suspension containing 0.2 wt% of the as-produced TEMPO-CNFs after heated in 180 °C saturated water vapor for 4, the heated TEMPO-CNFs became hydrogels.

Water is slowly vaporized from the heated aqueous suspension under ambient temperatures (18–28 °C) and obtained a yellowish film (this took 5–7 days). The yellowish substance was transferred to the solution after washing the film 3–5 times with an aqueous solution containing 70% ethanol (Fig. S3). The colorless, transparent films were used to measure their physiochemical properties by Raman, Fourier transform infrared spectroscopy (FT-IR), and XRD.

**Figure 3:**
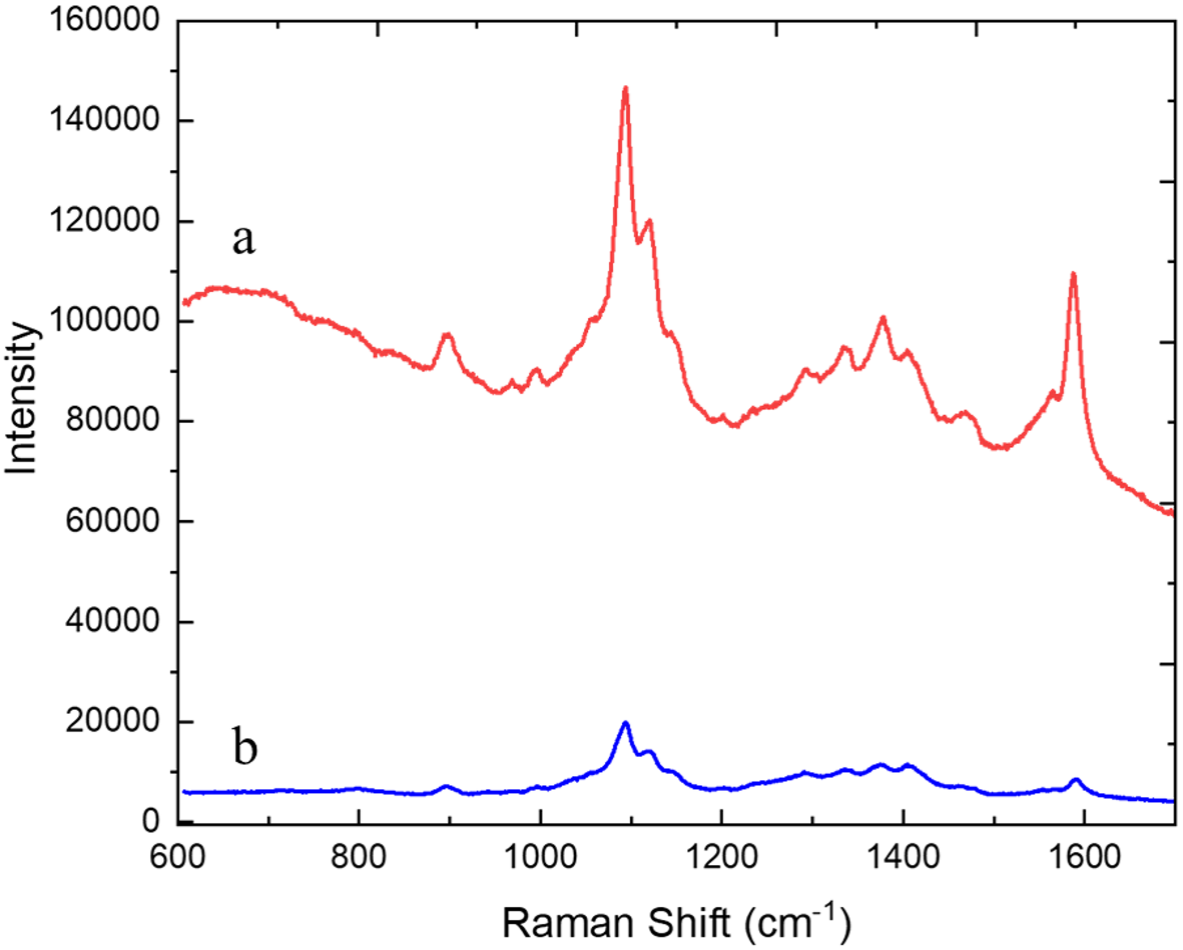
Raman spectra of TEMPO-CNFs after heated in 150 °C saturated water vapor for 4 h (a) or the as-produced TEMPO-CNFs (b). The excitation wavelength was 532 nm, exposure time 10 s, and laser power 74 mW with 6 accumulations. Thickness of the sample was about 120 μm by folding a 15-μm-thickness film 3 times.

Figure 3 shows the typical Raman spectra. Raman peaks corresponding to the skeletal vibrational modes of the asymmetric vibration of C–O–C at 1098 cm^−1^ (*v*_*as*_) and the symmetric vibration of C–O–C at 1120 cm^−1^ (*v*_*s*_) of the β (1–4) glycosidic linkage of the β-d-glucopyranosyl units^17–21^ of cellulose were clearly observed. A Raman peak corresponding to the stretching model of breathing vibrations of the glucopyranose rings (–C–C–) at 1153 cm^−1^^17–21^ was also observed. Raman peaks were also observed in the 1300–1500 cm^−1^ frequency region provide insights into the crystallinity and length/thickness of the multiple packed polysaccharide chains. The aqueous suspension used for preparing the film was the aqueous suspension containing 0.2 wt% of the as-produced TEMPO-CNFs after heated in 150 °C saturated water vapor for 4 h. The Raman spectra of the as-produced TEMPO-CNFs are also given in Fig. 3 for comparisons.

**Figure 4:**
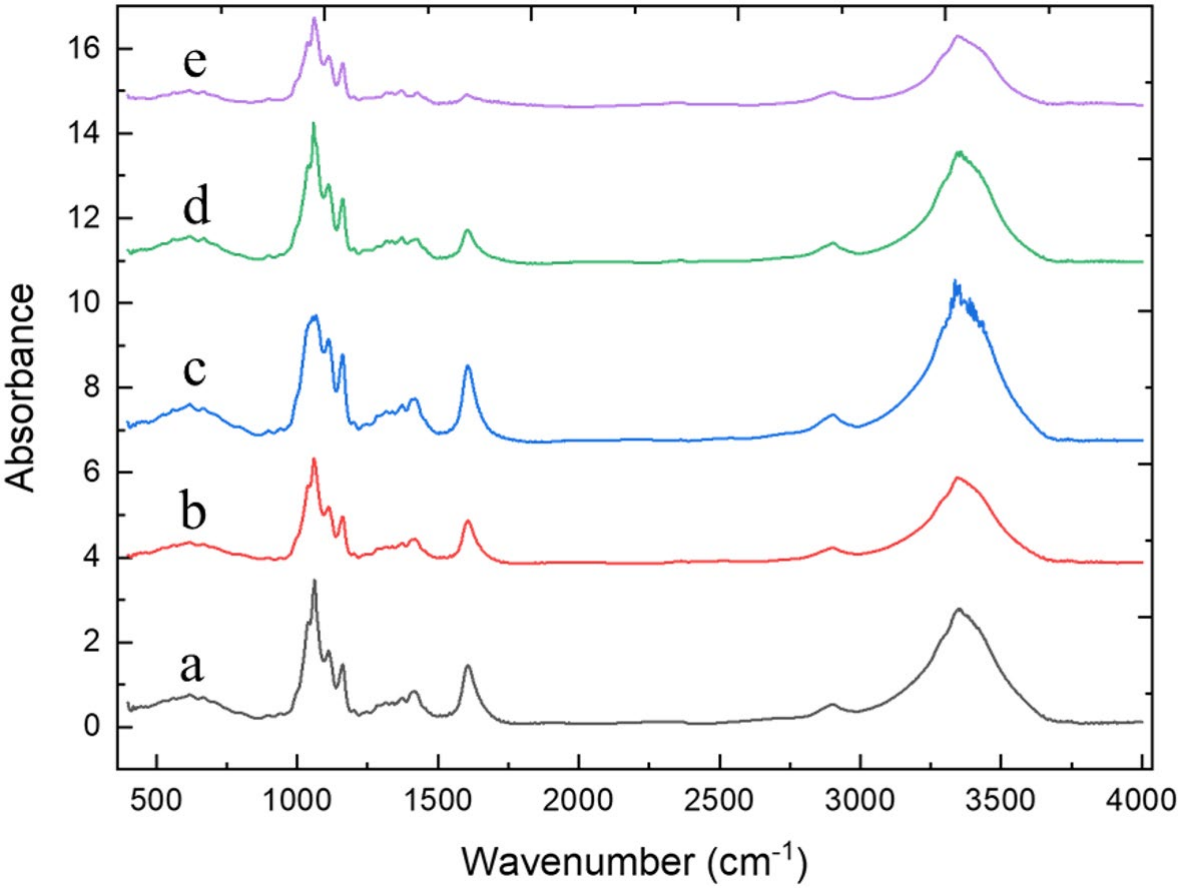
FT-RI spectrum of a 15-μm-thickness film sample made by using the as-produced TEMPO-CNFs (a) or the heated TEMPO-CNFs in 135 °C (b), 140 °C (c), 150 °C (d), or 160 °C (e) saturated water vapor, respectively, for 2 h. The film samples were measured directly (without folding).

The film samples were also characterized by FT-IR. Four film samples were prepared by using the aqueous suspension containing 0.2 wt% of the as-produced TEMPO-CNFs after heated at 135 °C, 140 °C, 150 °C, and 160 °C for 2 h, respectively, in saturated water vapor. Spectra identical to that of cellulose were observed^22–25^. Figure 4 shows the following spectra: 1100–900 cm^−1^, C–O–C stretching; 3600–3200 cm^−1^, O–H stretching; and the distinguishing peak of COO^−^ at 1600 cm^−1^. Notably, under the heating conditions of 160 °C/2 h, the intensity of the carboxylate peak (1600 cm^−1^) decreased sharply, indicating that the carboxylated polysaccharide chains situated on the outmost layers of TEMPO-CNFs had detached. The uncharged cellulose nanofibers exhibited a tendency to form hydrogels due to the external hydrogen bonding and the hydrophobic interactions.

Both the Raman and FT-IR spectra indicate that the short cellulose nanofibers are built up by multiple packed polysaccharide chains.

**Figure 5:**
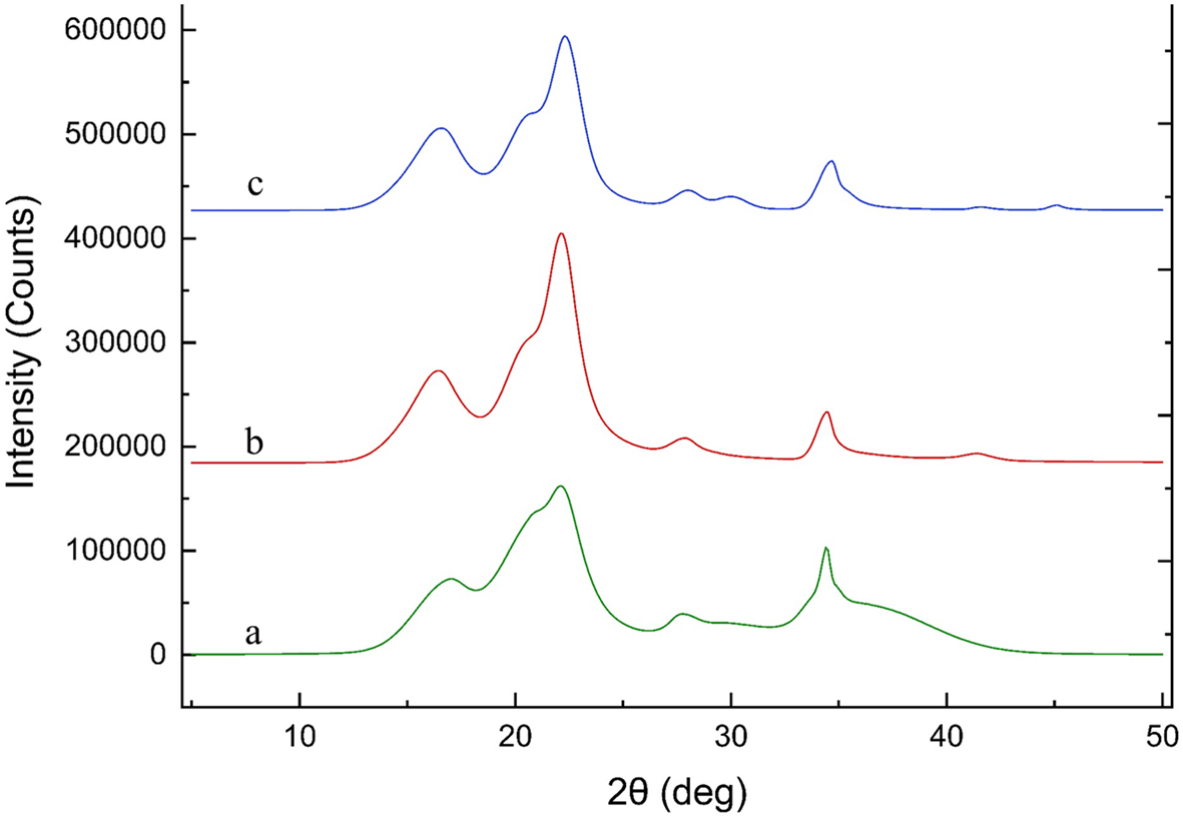
XRD analysis of the as-produced TEMPO-CNFs (a) and the as-produced TEMO-CNNFs after heated in 150 °C saturated water vapor for 4hours : sample (b) is with- and sample (c) is without-containing the yellowish substances. Thicknesses of the samples were about 120 μm by folding the 15 μm-thickness film samples 3 times.

TEMPO-CNFs changed drastically from longer nanofibers to shorter nanofibers in saturated water vapor under vapor pressures ≥ 0.2 MPs and temperature ≥ 120 °C. The drastic change in lengths led to a drastic change in the XRD patterns. XRD patterns of the as-produced TEMPO-CNFs were in consistent with both Iα and Iβ. XRD patterns of the heated TEMPO-CNFs, in contrast, were in consistent with Iβ. Figure 5 shows each of the corresponding XRD patterns. Details regarding the XRD patterns assignment are given in supporting information (Fig. S4). Table 1 summarizes the selected values, including 2 theta and the related full width at half-maximum (FWHM), and the d-values together with the Miller indices (h, k, l). Crystallite sizes and crystal/amorphous ratios of the as-produced TEMPO-CNFs and that of the heated TEMPO-CNFs are calculated based on Scherrer’s^26^ and Segal method^27^, respectively, also given in Table 1.

**Table 1:** Typical parameters selected from XRD measurements and the crystallite sizes, and the crystal/amorphous ratios of the as-produced TEMPO-CNFs and that of the heated-TEMPO-CNFs.

	2-Theta	FWHM	d-value (Å)	h	k	l	L* (nm)	C_am_ (%)**
As-produced TEMPO-CNFs	16.9	3.249	5.246	010/α; 110/β			2.50	61.1
	20.7	2.965	4.291	002/α; 102/β			2.75	
	22.2	1.953	4.004	− 110/α; 200/β			4.19	
	27.7	1.72	3.220	211/β			4.81	
	34.4	0.781	2.607	004/β			10.77	
Heated TEMPO-CNFs (with-containing the yellowish substances)	16.2	3.723	5.471	1	− 1	0	2.18	80.2
	16.6	1.756	5.340	1	1	0	4.62	
	20.7	2.758	4.291	1	0	2	2.96	
	22.2	1.484	4.004	2	0	0	5.52	
	27.9	1.512	3.197	2	1	1	5.47	
	34.4	0.867	2.607	0	0	4	9.71	
Heated TEMPO-CNFs (without-containing the yellowish substances)	16.3	3.757	5.438	1	− 1	0	2.16	80.4
	16.7	1.728	5.308	1	1	0	4.70	
	20.7	3.356	4.291	1	0	2	2.43	
	22.4	1.400	3.969	2	0	0	5.85	
	28	1.389	3.186	2	1	1	5.96	
	34.7	1.116	2.585	0	0	4	7.54	

*Crystallite sizes were estimated based on Scherrer equation (Eq. (1)).

**Crystal/amorphous ratios were calculated based on Segal method (Eq. (2) for Iα, or Eq. (3) for Iβ).

$$L=\frac{K\times\lambda}{\beta \times cos\theta}$$ (1).

$${C}_{am}=\frac{{I}_{002}-{I}_{am}}{{I}_{002}}$$ (2).

${C}_{am}=\frac{{I}_{200}-{I}_{am}}{{I}_{200}}$ (3), where in Eq. (1), θ, β, and λ are the Baragg’s angle, full width at half-maximum of the reflection, and wavelength of the X-ray source used, respectively; and in Eqs. (2) and (3), C_am_ denotes the crystal/amorphous ratios, I_002_ and I_200_ denote the maximum intensities of diffractions of 002 (in case of Iα), and 200 (in case of Iβ), respectively, and I_am_ is the maximum intensities of the diffraction of amorphous (2θ = 18.4). The widths were estimated to be 4.19, 5.52, and 5.85 nm, for the as-produced-TEMPO-CNFs, the heated TEMPO-CNFs with- and without-containing the yellowish substance, respectively, based on the XRD data.

Notably, the heated TEMPO-CNFs showed higher crystal/amorphous ratios. The ratios were 61.1%, 80.2% and 80.4%, for the as-produced TEMPO-CNFs, the heated TEMPO-CNFs with-and without-containing the yellowish substances, respectively. The increase in crystallinities had elevated hydrophilicities. The hydrophilicities were estimated based on the ability to stabilize oil/water emulsions prepared with the as-produced TEMPO-CNFs or the heated-TEMPO-CNFs as the stabilizers (Fig. S5).

From our experimental observations, it is reasonable to assume that the native plant cellulose microfibrils are not seamlessly long nanofibers, but are “jointed structures”.

**Figure 6:**
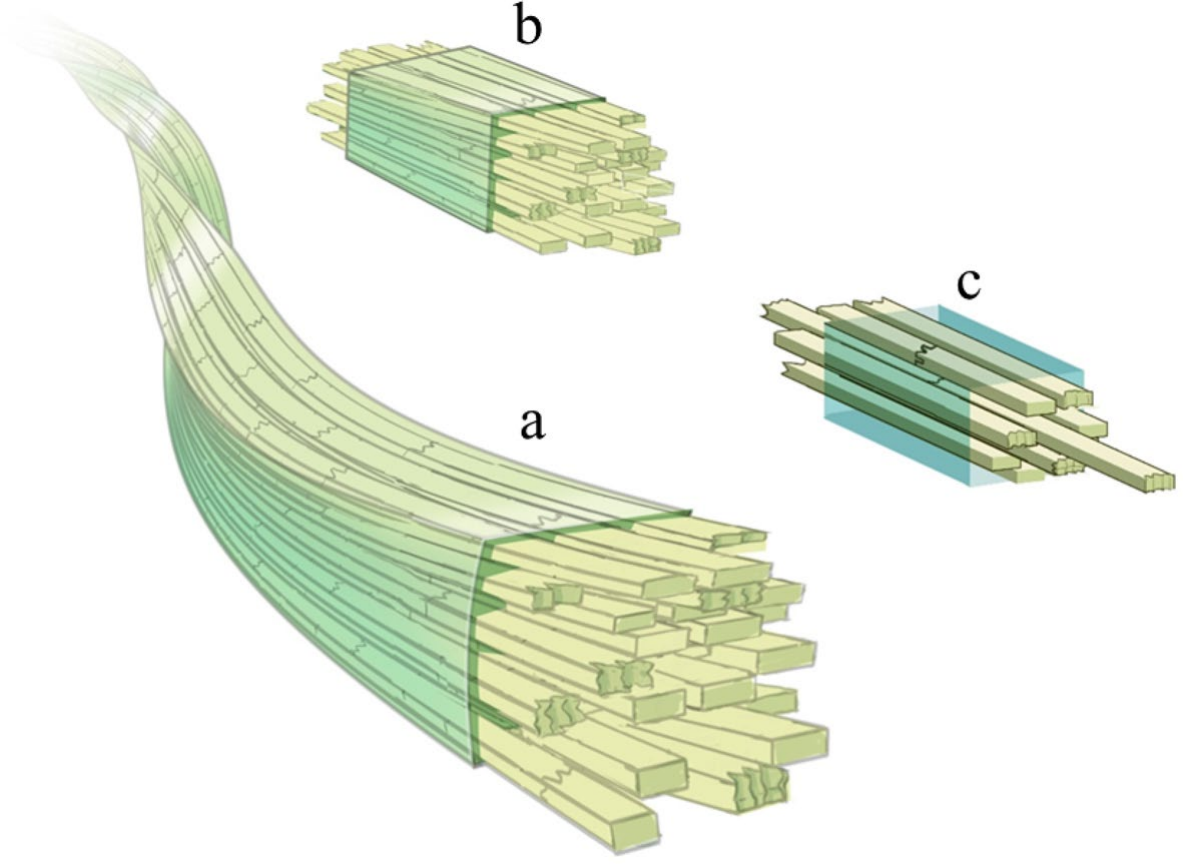
Finger-jointing structures for lengthening cellulose microfibrils. (**a**) Polysaccharide chains in cellulose microfibrils are not the seamlessly long length of polymers, but relatively short (10 s to 100 s of nm in length), and each of the short chains is jointed in a finger-jointing manner. Finger-jointing is weaker but flexible compared to the packed (vertical) structures. Under tough conditions, such as in saturated water vapor at higher temperature/pressure, cellulose microfibrils unzipped into short blocks starting at the finger-jointing boundaries. (**b**) Once the microfibril unzipped into short blocks, (**c**) polysaccharide chains situated at the outmost layers of the block detached from the main body as a thinner block.

We propose a finger-jointing model, as shown in Fig. 6, to describe the possible ultrastructures of the native plant cellulose microfibrils. Key concepts of this newly proposed model are summarized as follows:

(i) the native plant cellulose microfibrils are not seamlessly long structures, but are combined and jointed structures composed of many shorter cellulose blocks that are jointed in a simple jointing manner;
(ii) finger-jointing is the most possible manner by which the short cellulose blocks were jointed in to longer lengths of cellulose microfibrils;
(iii) polysaccharide chains of the short cellulose blocks organized in Iβ manners but the best positions for each chains left an uneven edge which is capable of jointing another short cellulose block in a serial jointing manner via finger-jointing;
(iv) the jointed regions can be either Iα or amorphous, depending on the positions and the distances among the polysaccharide chains jointed in proximity;
(v) the jointed structures enable cellulose microfibrils to twist, to curve, and to be tolerant toward physical fatigues;
(vi) the jointed regions are much weaker than the Iβ based blocks, in other words, the jointed structures have largely or completely been destroyed under saturated water vapor, but the Iβ basic structures remain unchanged;
(vii) cellulose synthases shall be on the edges of each polysaccharide chain so that the newly synthesized polysaccharide chains can be naturally jointed;
(viii) from the biosynthesis point of view, plants are not working all day for biosynthesis of cellulose, but working and resting rhythms may also exist in the plant world.

## Conclusions

Plant cellulose can be key to achieving the goals of sustainable development goals (SDGs). Nevertheless, the ultrastructures regarding the native plant cellulose and how their ultrastructures relate to their macro-functions remain largely unknown. We have much to learn about the length of each polysaccharide chain, the degree of orientation, and the intensity of interactions among the packed polysaccharide chains and, the ultrastructures of the cellulose microfibrils. In a previous study, Li and Renneckar^28^ showed that TEMPO-CNFs can be split down to a single or double digit Angstrom thickness with 100 s of nanometers in length under extensive ultra-sonication. This experimental observation also suggests that cellulose microfibrils are not seamlessly long-structured substances. In other words, plants are not working all day to synthesize cellulose, but a working and resting rhythm exists in plants, resulting in jointed bio-products. Other bio-nanostructures, such as DNA and proteins, may also be a category of jointed structures and could be unzipped into basic (building) blocks once the hydrogen binding interactions among the basic blocks are weakened and/or destroyed. In conclusion, jointing of a certain number of short-length building blocks is a simple yet efficient manner by which the giant-sized bio-structures with specified shapes and diverse functionalities are established.

## Methods

TEMPO-CNFs were purchased from Dai-ichi Kogyo Seiyaku Co. Ltd. (Kyoto, Japan); they were produced via TEMPO oxidation of native plant cellulose. The as-received suspension was diluted to 0.2 wt% with deionized water. The resulting suspension containing 0.2 wt% of the as-produced TEMPO-CNFs was used throughout this study. Sealed thermal decomposition vessels were obtained from San-ai Kagaku Co., Ltd. (Nagoya, Japan) and used for heating the aqueous TEMPO-CNF suspensions. The thermal decomposition vessels consisted of a sealed PTFE-based inner vessel and a sealed steel-based outer cylinder. Approximately 50 mL of the aqueous suspension containing 0.2 wt% of the as-produced TEMPO-CNFs was sealed in the PTFE vessel and the sealed vessel placed in an electric heating oven for a predetermined time at a specified temperature. After the sealed vessel was cooled down to room temperatures, the heated aqueous suspension was removed from the vessel and used to characterize their physiochemical properties. To prepare the film, approximately 15 g of the heated suspension was placed in a polystyrene-based petri dish and water vaporized slowly under ambient temperatures (this took 5–7 days), obtaining a yellowish film. The yellowish substance was removed by washing the film sample with an aqueous solution containing 70% ethanol, resulting in a transparent film. The film sample was dried at a constant temperature of 40 °C overnight and then used for characterization via Raman spectrometry, FT-IR, and XRD.

Both the as-produced TEMPO-CNFs and the heated TEMPO-CNFs were analyzed via Raman spectrometer^17–21^ and FT-IR^22–25^. The Raman spectrometer was Renishaw (inVia Raman) with four excitation wavelengths: 488 nm, 532 nm, 633 nm, and 785 nm. The Raman measurements were performed mainly with excitation at 532 nm (exposure time 10 s, laser power 74 mW, 6 accumulations). The FT-IR spectra were obtained using a Jasco FT-IR-460 Fourier Transform Infrared Spectrometer.

Both the as-produced TEMPO-CNFs and the heated TEMPO-CNFs were also analyzed via XRD^29–31^. The XRD was a Rigaku Smart Lab X-Ray Diffractometer with Cu-Kα radiation (λ = 1.5418 Å) operating at 40 kV and 30 mA, a scanning speed of 0.5 min^−1^, and a range (2θ) of 5°–50°. Thickness of samples was ~ 120 μm (by folding the film sample 3 times).

The atomic force microscope (AFM) used throughout this study was a Shimadzu scanning probe microscope/atomic force microscope SPM-9700HT (Kyoto, Japan), operating at tapping mode with resolutions of XY 0.2 nm and Z 0.01 nm. The heated aqueous suspension of 0.2 wt% TEMPO-CNFs was diluted about 1/1000 with 80% ethanol or deionized water. Approximately 1 μL was deposited on a silicon wafer (plate) and then dried under ambient temperatures before analysis.

We directly analyzed both the as-produced TEMPO-CNFs and the heated-TEMPO-CNFs via JEM-2100F. The 0.2 wt% TEMPO-CNF suspension was diluted about 1/1000 with 80% ethanol or deionized water, the diluted suspension was deposited on carbon-coated electron microscope grids (u1017-5 nm) and then dried under ambient temperatures before analysis. The sample holder was under cooling via nitrogen during the time of analysis. We analyzed the negatively stained TEMPO-CNFs (both the as-produced and the heated TEMPO-CNFs) via JEM-2800. We used a 2% uranyl acetate solution to negatively stain TEMPO-CNFs^32^. Both JEM-2100F and JEM-2800 were the JEOL products (JEOL, Tokyo, Japan). The analysis was operated at voltages of 200 kV.

## Supplementary Information

Supplementary Information.
