# Supplementary material for: A finger-jointing model for describing ultrastructures of cellulose microfibrils
Source: Sci Rep. 2021 May 12;11:10055. doi: 10.1038/s41598-021-89435-6 (PMC8115659; doi:10.1038/s41598-021-89435-6)
Supplement: Supplementary file 1 — Supplementary Information. [file 41598_2021_89435_MOESM1_ESM.docx]

**Supporting information:**

**A finger-jointing model for describing ultrastructures of cellulose microfibrils**

Bunshi Fugetsu1, Vipin Adavan Kiliyankil, Shoichi Takiguchi, Ichiro Sakata, Morinobu Endo


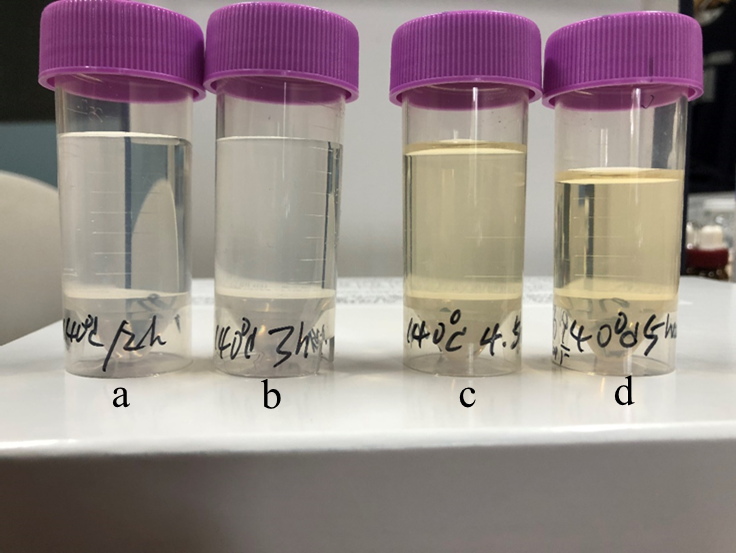


Figure S1. A photo of aqueous suspensions containing 0.2 wt% of the as-produced TEMPO-CNFs after heated in 140°C saturated water vapor for 2 h (a), 3 h (b), 4.5 h (c), and 5 h (d), respectively.


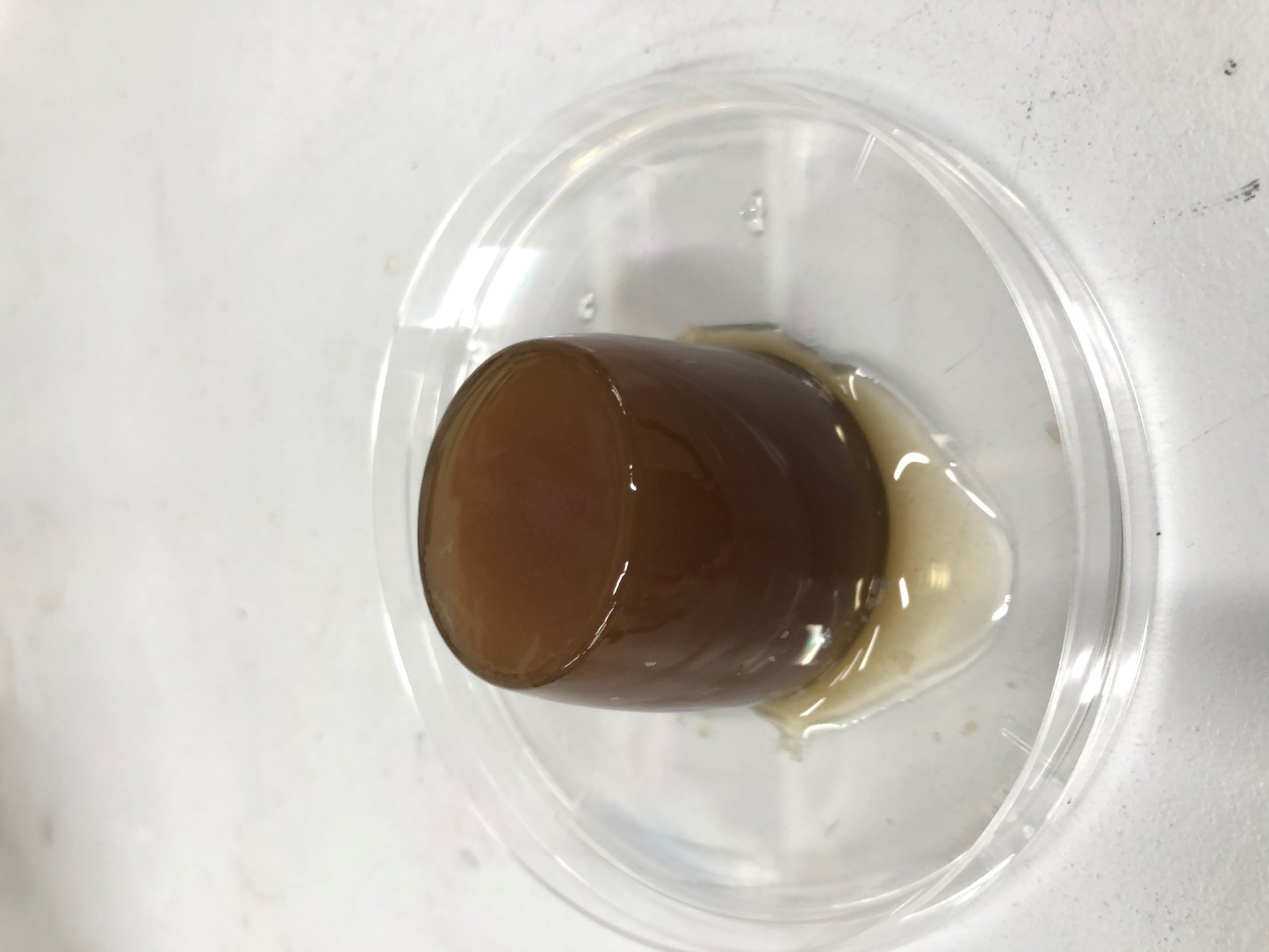


Figure S2. A photo of an aqueous suspension contained 0.2 wt% of the as-produced TEMPO-CNFs after heated in 180 °C saturated water vapor for 4 h. The heated TEMPO-CNFs became hydrogels.


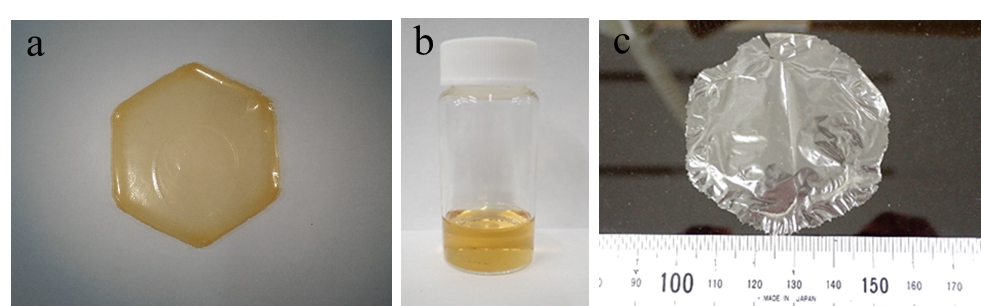
Figure S3. Preparation of film samples for observation of characteristic peaks via Raman, FT-IR and XRD. (a) Water vaporized and obtained a yellowish film. (b) The yellowish substance was removed by washing the film with an aqueous solution containing 70% ethanol. (c) After drying the washed film at 40°C overnight, we obtained colorless, transparent film samples.


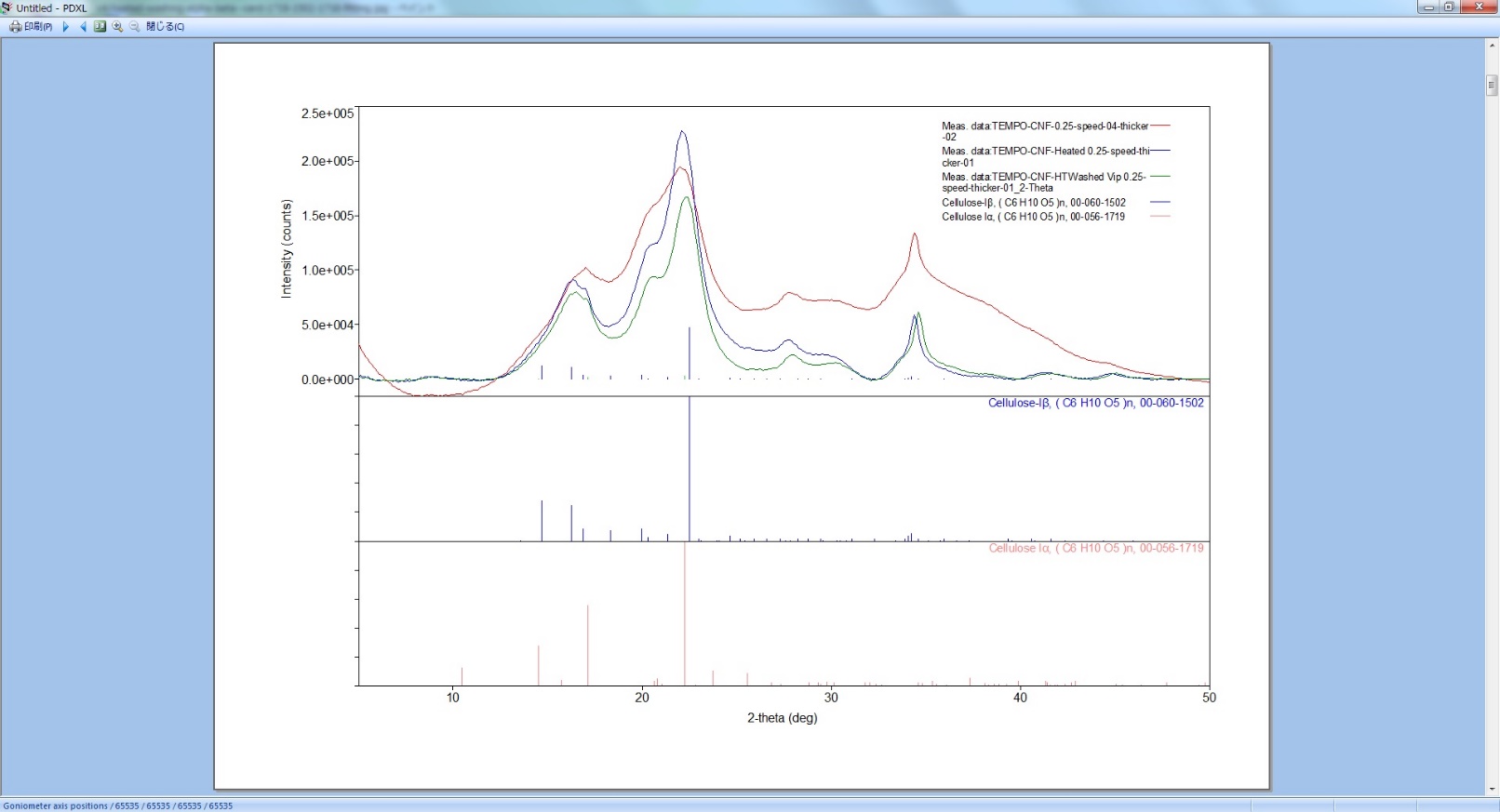


Figure S4 Peak assegment based on data base on cellulos Iα and cellulose Iβ aquoted from Card No. 00-056-1719 and card No. 00-060-1502. XRD patterns of the as-produced TEMPO-CNFs (the red line) were in consistent with both cellulose Iα and Iβ. XRD patterns of the heated-TEMPO-CNFs with-containing (the blue line) and without-containing (the green line) the yallowish substances were in sonsistent with cellulose Iβ.


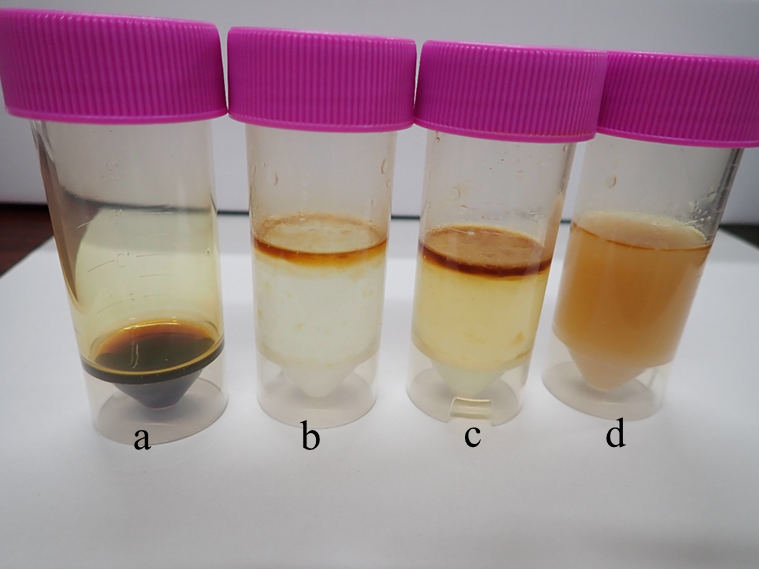


Figure S5 Properties of the as-produced TEMPO-CNFs and that of the heated TEMPO-CNFs (heated in 150 °C saturated water vapor for 4 hours) on stabilization of oil/water emulsions. Coffee essential oil (a) was added in deionized water (b), in the aqueous suspension containing 0.2wt% of the heated TEMPO-CNFs (c), and in the aqueous suspension containing 0.2 wt% of the as-produced TEMPO-CNFs (d) at a ratio of 1/50 (v/v) and was then well-mixed under ultra-sonication and oil/water emulsions formed. This photo was the above mentioned emulsions after past a 3-days of stand-still. Coffee essential oil self-isolated from the deionized-water/oil and the heated-TEMPO-CNF/oil emulsions; the as-produced-TEMPO-CNF/oil emulsions remained stable. Thus, the heated TEMPO-CNFs are much hydrophilic than the as-produced TEMPO-CNFs.
